# A new urethral catheterisation device (UCD) to manage difficult urethral catheterisation

**DOI:** 10.1007/s00345-018-2499-9

**Published:** 2018-09-24

**Authors:** S. Bugeja, K. Mistry, I. H. W. Yim, A. Tamimi, N. Roberts, A. R. Mundy

**Affiliations:** 1 Urology Department, University College London Hospitals NHS Foundation Trust, London, UK; 2 The Heart Hospital, University College London Hospitals NHS Foundation Trust, London, UK

**Keywords:** Urethral catheterisation device, Difficult urethral catheterisation, Urethral catheterisation injury, Male catheterisation algorithm, Urethrotech

## Abstract

The cost of urethral catheterisation injury (UCI) is significant, but the true incidence of patient care error is difficult to establish in the absence of specific hospital codes recording difficult urethral catheterisation (DUC) and UCI. For many years urologists are familiar passing a non-traumatic hydrophilic guidewire blindly into the bladder to aid urethral catheter insertion in difficult circumstances. However, so far, no purpose-built regulated medical device was available on the market and clinicians had to improvise. Urethrotech filled that gap and developed the Urethral Catheterisation Device (UCD^®^), which integrates a standard hydrophilic Nitinol guidewire into a 3-way 16F Silicone urethral catheter design to enable safe second-line urethral catheterisation when first-line catheterisation with a standard urethral catheter is unsuccessful. The safety and efficacy of UCD^®^ catheterisation were evaluated in consecutive cohorts of men undergoing cardiac surgery and compared to the incidence of DUC and UCI with standard Foley catheterisation. A simple new Male Catheterisation Algorithm is proposed that can deliver a safe male urethral catheterisation treatment protocol for all clinical settings of healthcare services, which is easy to implement and integrate into standard catheterisation training programs to manage DUC and avoid UCI, empowering a frontline workforce to deliver better patient care.

## Introduction

Urethral catheterisation is one of the commonest procedures performed both in the community and hospital setting. Approximately 25% of the patients admitted to hospital will have a catheter at some point during their stay [1] and 7% of nursing home residents are managed by long-term catheterisation. Foley catheters form about 70% of the market and in the United States about 30 million Foley catheters were sold in 2017.

In the United States, nursing staff perform the majority of catheterisations for both male and female patients with well-established procedure guidelines with particular focus on reducing catheter-associated urinary tract infections (CAUTI’s). Although difficult urethral catheterisation (DUC) occurs only in a small percentage of urethral catheterisations overall, it is an important problem due to the number of catheterisations that take place on a daily basis throughout the Healthcare Service. Often, multiple catheterisation attempts are made, ranging from 1.6 to 3.2, before the patient is referred to Urology services causing significant urethral catheterisation injury (UCI) in 32% of men [2]. Hence, when DUC does occur, the catheterisation emergency can easily escalate out of control, leading to acute UCI with bleeding requiring hospital admission for more invasive specialist procedures. On the other hand, DUC can delay patient care with the risk of cancellation of other remaining planned surgical procedures when pre-operative catheterisation is mandatory.

Published data on UCI are limited probably due to the absence of specific hospital codes for de-facto procedure complications and the true incidence of DUC and UCI is difficult to establish. Over 1 year within a tertiary teaching hospital, 6% of all urological referrals were related to complications arising from male urethral catheterisation [3]. A calculated incidence of 0.7 iatrogenic urethral catheter injuries per 1,000 adult male hospital admissions was reported in another single academic tertiary hospital [4] and other investigators report UCI to be as common as symptomatic urinary tract infection [5].

The cost to treat UCI is about $10,000 per case based on a reported UCI incidence of 6.7 per 1000 catheters inserted [6]. This yields a total healthcare burden in the US of $2 billion dollars per year. These costs are exclusive of managing long-term complications of urethral strictures or any potential medico-legal costs [7].

Every hospital department and clinical area where urethral catheterisation takes place has to deal with the unpredictable problem of DUC. Clinical areas where such events occur more frequently are ER/OR departments, care of the elderly and Palliative care/Nursing homes.

Technological advances in the urethral catheter design to solve the problem of DUC were in demand for decades [8], thus Urethrotech has developed a purpose-built Urethral Catheterisation Device (UCD^®^) for second-line urethral catheterisation based on the well-established clinical manoeuvre of passing a tube over a guidewire (Seldinger Technique) [9]. Indeed, for years urologists have been using this Seldinger principle with varying improvised guidewire-catheterisation techniques [10–14], which may pose a sharps injury risk apart from violating the integrity of the urethral catheter design.

We herewith describe the experience with the new Urethrotech UCD^®^, which was evaluated and compared to a standard catheter performance in a matched cohort of men undergoing cardiac surgery. We propose a simple new algorithm for male catheterisation in view to prevent UCI.

## Materials and methods

**Fig. 1:**
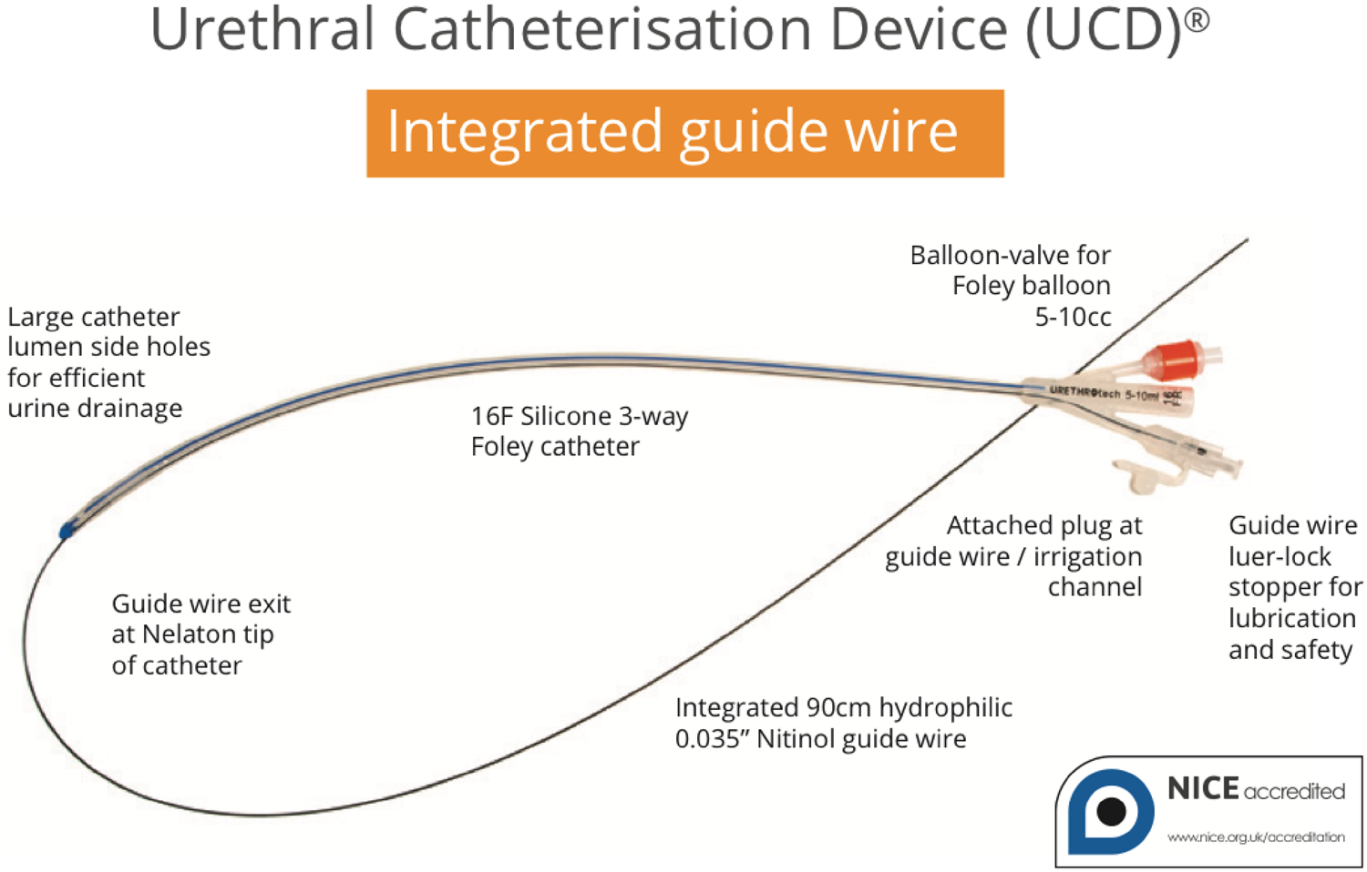
Urethrotech Urethral Catheterisation Device (UCD^®^)

**Fig. 2:**
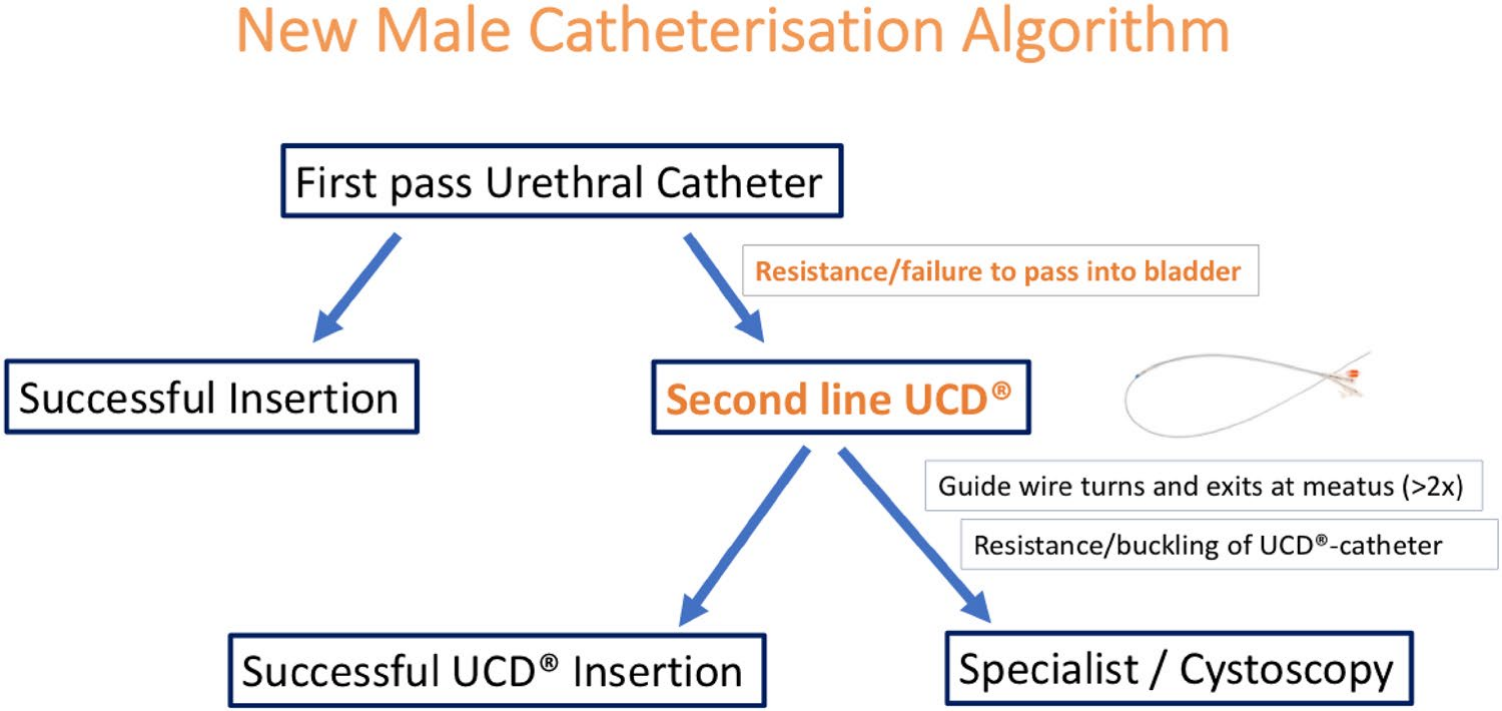
New Male Catheterisation Algorithm to manage difficult urethral catheterisation

The Urethrotech UCD^®^ integrates a standard hydrophilic Nitinol guidewire into a 16F 3-way Silicone Foley catheter (Fig. 1). The single-use sterile medical device has a 5-year shelf-life and can readily be stocked on any Urethral Catheterisation Trolley (UCT). Once the guidewire is lubricated with sterile water or saline, the device is ready to use. The intended use of the UCD^®^ is second-line urethral catheterisation when resistance is encountered during first-line standard urethral catheterisation (Fig. 2).

Because the UCD^®^ guidewire and catheter form one unit, it’s very easy to pass the guidewire into the bladder where it curls up and automatically drags the catheter behind without getting stuck in an enlarged prostate, which is the most common problem of DUC in the elderly.

Once the catheter shaft is inserted fully to the hub and urine drains freely [15], the Foley balloon is inflated after which the guidewire is removed. The attached UCD^®^ plug closes the guidewire channel, or one could start bladder irrigation (or take a direct bladder urine specimen) through the same channel.

The main safety feature of the UCD^®^ is the material characteristic and length of the integrated UCD^®^ hydrophilic Nitinol guidewire. Should the guidewire fail to pass into the bladder and turn around repeatedly to exit at the urethral meatus, the Instruction For Use (IFU) instructs the healthcare professional to abandon the procedure. The UCD^®^ guidewire will do no harm on its own. The second-line catheterisation procedure should also be abandoned if resistance is encountered again during catheter advancement. Such resistance is likely caused by an iatrogenic urological problem, such as bladder neck contracture after radical prostatectomy or a dense urethral stricture, and the patient requires a diagnostic cystoscopy by a urologist for the management of the underlying cause of the problem.

In this prospective observational cohort study, the safety and efficacy of UCD^®^ catheterisation were evaluated and compared to a standard Foley catheterisation cohort in men undergoing cardiac surgery in a single cardiothoracic centre. Institutional review board-approval for the evaluation was granted and informed consent was obtained from all individual participants included in the study. The potential complication risk from UCI in this cardiac surgery cohort was considerable, in view of the fact that all men have to be fully anticoagulated at the end of their cardiac procedure (bypass or valve replacement), creating a surgical management nightmare if urethral bleeding from traumatic catheterisation would occur. In group A, 74 consecutive male patients (mean age 66.8 years) underwent standard catheterisation using a 16F Silicone Foley catheter. In group B, 100 consecutive male patients (mean age 67.2 years) underwent urethral catheterisation using the UCD^®^.

In both cohorts, the catheterisation was performed after the patient was anaesthetised by theatre nurses trained in standard Foley and UCD^®^ catheterisation. Data on ease-of-use, adverse events and catheter-related symptoms after the procedure were collected prospectively for both cohorts.

To substantiate the incidence of difficult/traumatic urethral catheterisation, a retrospective audit of 150 consecutive men undergoing cardiothoracic surgery in the same unit was also performed.

## Results

127 of 150 patients reviewed retrospectively had complete documentation in their clinical notes but none had been consented or counselled about potential urethral catheterisation complications. 4 of these patients (2.7%) required suprapubic catheterisation for traumatic or unsuccessful urethral catheterisation.

**Fig. 3:**
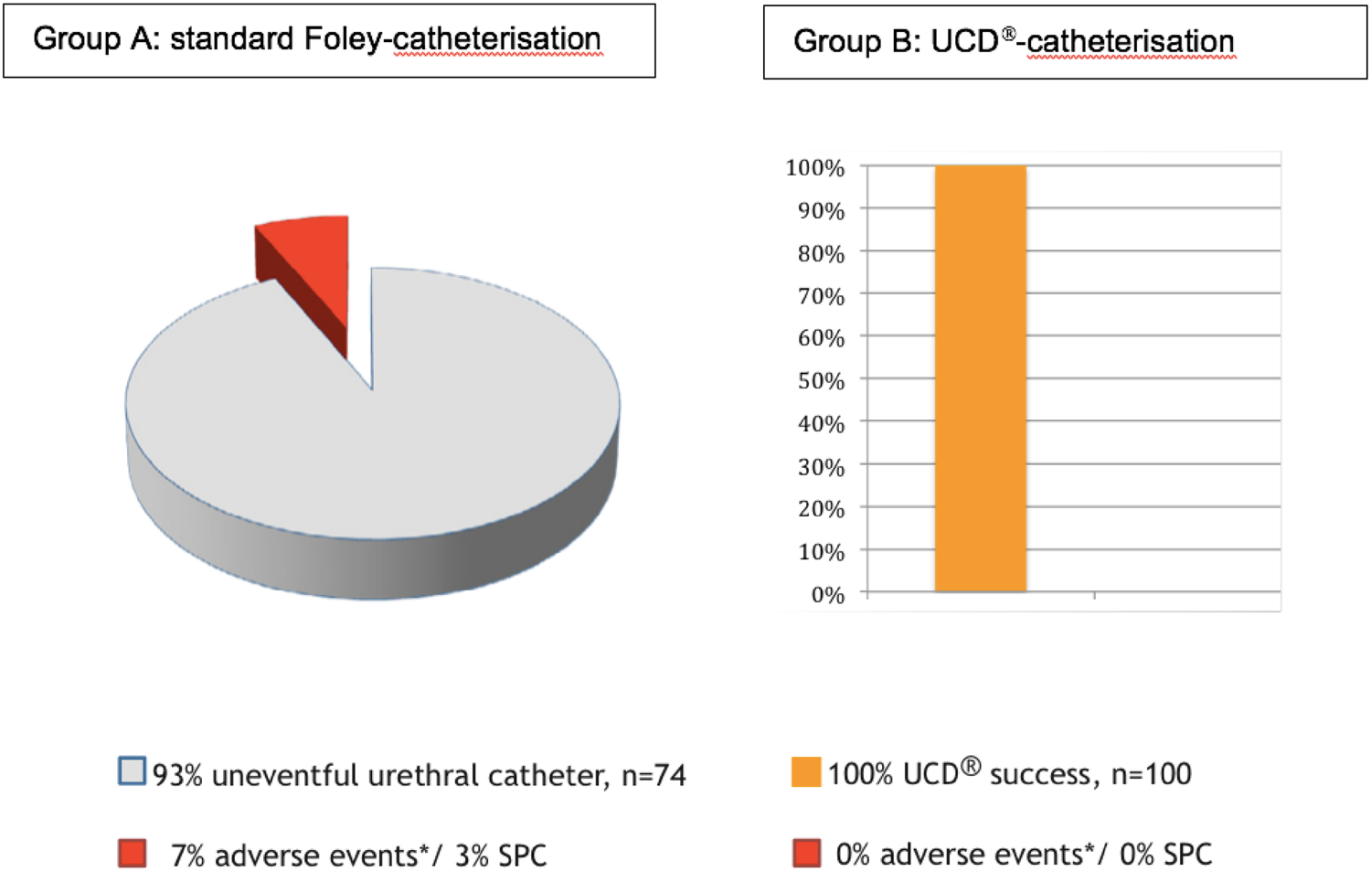
Urethral catheterisation adverse events. Group A: standard Foley catheterisation; group B: UCD^®^ catheterisation

In prospective group A, of the 74 men undergoing standard Foley catheterisation, 7 (9%) experienced some form of acute or late adverse event related to the catheterisation procedure: 5 (7%) suffered demonstrable acute adverse events of which 3 (4%) had significant urethral bleeding, and 2 (3%) patients required suprapubic catheterisation because it wasn’t possible after repeated attempts to insert the catheter into the bladder (Fig. 3). These 2 patients were later found to have an enlarged prostate as the cause of failed catheterisation. Another 2 patients reported ongoing complaints suspected to be related to the catheterisation procedure; 1 suffered from urethral pain for one month after the procedure and the second complained of chronic pain in the perineum (to investigate this further was not scope of this study and hence those 2 patients were excluded from the graph in Fig. 3).

In group B, all 100 patients undergoing UCD^®^ catheterisations were catheterised uneventfully first-pass. They did not experience any adverse events during the catheterisation procedure itself or in the post-operative period. Healthcare professionals performing UCD^®^ catheterisation had no problems using the new device and liked the ease-of-use and minimal variation from standard catheterisation.

## Discussion

Urethral catheterisation injury (UCI) converts a relatively simple procedure into an emergency with potential long-term patient morbidity and significant financial burden to both patient and healthcare service, in extremis leading to potentially fatal complications of urosepsis and Fournier’s gangrene.

Acute UCI complications such as bleeding and false passage traditionally require catheterisation under cystoscopic vision or suprapubic catheter insertion. This usually results in unnecessary hospital admission and prolonged hospital stay.

The cost of managing UCI was €335,377 ($371,790) during a 6-month study period at two hospitals in the UK. 81% sustained complications Clavien-Dindo grade 2 or greater and the additional length of hospital stay was 9.4 ± 10 (2-53) days [6]. These costs did not include management of long-term complications such as urethral stricture disease. In addition, UCI is the commonest catheter-related malpractice claim [7].

Several studies have shown a significant reduction of UCI after appropriate training with a fivefold incidence reduction from 3.2/1000 to 0.7/1000 [3]. Nevertheless, despite appropriate training, the very fact that an enlarged prostate can cause distortion of an otherwise straight urethral path into the bladder makes it equally impossible to pass a standard catheter, irrespective of the eminence of the operator. The vast majority of DUC-related urological consultations are not due to an underlying luminal constriction, i.e. urethral stricture, but when urethral trauma has already occurred in the presence of non-constricted but problematic anatomy such as in benign hyperplasia of the prostate (BPH); or in younger men, the presence of a tight external sphincter due to anxiety [16].

DUC or UCI is reported to occur between 0.3 and 3% of urethral catheterisations [17] but as high as 32% [2]. The main reason for this wide variation is a lack of hospital episode codes for DUC, which makes accurate assessment of UCI prevalence near impossible.

The commonest specialist approach to solve DUC is the insertion of the urethral catheter over a guidewire [16]. This railroading technique was described by Seldinger in 1953 [9]. It is therefore not surprising that this technique was recommended for DUC as well [18]. Urologists like to place guidewires under direct vision using a cystoscope. However, this is not always a feasible solution since DUC is encountered in every clinical environment including community care, where urological equipment and expertise is not readily available and the patient would need to be referred to a hospital.

Since the Terumo^®^ guidewire came to market in the mid-1980s, it is known to urologists that this non-traumatic hydrophilic Nitinol guidewire can be placed safely into the bladder without vision. The technique of blind guidewire insertion has been described initially by Freid and Smith [19]. Various improvised ‘Do-It-Yourself’ guidewire catheterisation techniques have since been described [10–14]. Some thread the guidewire through one of the eye of the catheter, some cut off the tip, and others make a hole at the catheter tip with a needle or knife to thread the guidewire through; however, in doing so, risk sustaining a sharps/needle-stick injury or damaging the balloon inflation channel.

Urethrotech^®^ designed a long overdue innovative urethral catheter design to provide frontline staff with a readily available, sterile and properly regulated consumable to manage DUC, which can easily be stocked on any UCT to provide safe second-line catheterisation without the need for specialist equipment, suitable for all healthcare professionals who otherwise place urethral catheters. The pragmatic advantages of the Urethrotech UCD^®^ are many: having guidewire and catheter integrated as a single unit means that DUC can now be managed single handedly at the patient’s bedside. All it takes to activate the guidewire is a syringe of water! Hence, if used in the community, the UCD^®^ could avoid unnecessary referrals to hospital to manage DUC. On top, because the UCD^®^ guidewire runs in the wall of the catheter, the urine drainage channel is kept empty and can drain urine freely confirming correct placement of the catheter in the bladder (apart from the fact that community nurses prefer to attach the urine bag before catheter placement to avoid urine spillage into the patient’s bed). Once the guidewire is the removed after safe UCD^®^ catheter placement, the guidewire channel could be used for irrigation or urine specimen collection.

The safety and efficacy of the new Urethrotech UCD^®^ were deliberately evaluated in high-risk patients undergoing elective cardiac surgery with a reported UCI incidence of 4% [20]. Any form of urethral trauma puts these patients at high risk of bleeding and its management may compromise their surgical outcome. Cardiothoracic surgery often takes place in specialist hospitals without immediate access to urological support, as in our study. We recorded DUC with demonstrable adverse events in 5(7%) of the patients catheterised with a standard catheter (group A). Three (4%) had bleeding and two (3%) required risky suprapubic catheterisation. However, patients subsequently undergoing UCD^®^ catheterisation (group B) were all successfully catheterised by theatre nursing staff with no adverse events and high user satisfaction.

The Urethrotech UCD^®^ is also useful after specialist urology procedures [21] and was successfully implemented in our nurse-led TWOC clinic [22, 23]. The National Institute for Health and Clinical Excellence (NICE) in the UK has accredited the Urethrotech UCD^®^ as a cost-effective second-line catheterisation solution to manage DUC [24].

We herewith propose a simple new Male Catheterisation Algorithm (Fig. 2) that can deliver safe male urethral catheterisation for all healthcare services, which is easy to implement and integrate into standard catheterisation training programs to manage DUC and avoid UCI.

## Conclusion

The Urethrotech UCD^®^ provides a ready-to-use sterile medical device for safe second-line urethral catheterisation to manage DUC without the problems associated with various improvised techniques. The new Male Catheterisation Algorithm rationalises specialist time and resources (flexible cystoscopy) when clinically necessary which is cost-effective in its own right. This prospective safety and efficacy evaluation study demonstrated that the Urethrotech UCD^®^ is successful and easy to use even in high-risk patients. Moreover, second-line UCD^®^ catheterisation is easy to implement in many different clinical settings with the potential of reducing unnecessary Accident and Emergency department attendance and hospital admissions. The UCD^®^ empowers a comprehensive nurse-led male catheterisation service. Avoiding UCI in the first place is not only better patient care but inevitably cost-effective.

## Abbreviations

CAUTI: Catheter-associated urinary tract infection
DUC: Difficult urethral catheterisation
IFU: Instruction for use
UCI: Urethral catheterisation injury
UCD^®^: Urethral catheterisation device
UCT: Urethral catheterisation trolley
